# PromptSE: drug side effect prediction with LLM-derived pharmacological representations

**DOI:** 10.1038/s41598-026-55667-7

**Published:** 2026-05-30

**Authors:** Yuqing Xia, Hao Wang, Tianyi Li, Yilong Niu

**Affiliations:** School of Data Sciences, Zhejiang University of Finance and Economics, Hangzhou, 310018 China

**Keywords:** Computational biology and bioinformatics, Drug discovery, Mathematics and computing

## Abstract

Predicting drug-side effect associations is vital for drug discovery and patient safety. Accurate prediction requires high-quality representations of both drugs and side effects. While drug representations have advanced due to rich structured data, side effect information is mostly unstructured, symptom-oriented and heterogeneous texts, making it hard to capture underlying pharmacological mechanisms. We propose prompt-based side effect prediction (PromptSE), a hybrid framework that combines the reasoning power of large language models with the predictive capability of deep learning. PromptSE employs stepwise prompting tailored to the characteristics of side effect texts, moving beyond simple encoding or conventional prompting, to generate pharmacologically relevant representations. These representations are then fed into a deep learning module for drug-side effect prediction. Building on this framework, PromptSE+ extends the prediction module by integrating multi-modal drug information. Rare entity representations are further refined via a custom graph neural network module. Experiments show that PromptSE outperforms non-drug-informed baselines by 9.26% in AUPR, confirming the effectiveness of our representations. PromptSE+ enhances state-of-the-art drug-side effect prediction methods across all metrics, including a 1.81% AUPR gain, demonstrating its compatibility with advanced methods and potential to support safer drug development and more reliable pharmacological research.

## Introduction

Side effects are unintended responses to a therapeutic dose of a drug beyond its intended therapeutic effects^1^. They pose a significant challenge in healthcare, ranking as the fourth leading cause of mortality after cardiovascular disease, cancer, and infections^2^. Identifying drug-side effect associations is critical but challenging, as traditional biological and pharmacological experiments are resource-intensive and time-consuming^3,4^, underscoring the need for computational models^5^. Computational models frame the task as a binary classification problem: learning a mapping from drug-side effect pairs to a prediction of their association likelihood^6^. Accurate binary classification depends on the quality and discriminability of drug and side effect representations. Ideally, effective representations should map side effects caused by common drugs and drugs that cause common side effects close together, while keeping unrelated side effects and drugs apart.

Drug representation learning has advanced significantly, leveraging diverse data^7^ such as chemical substructures^8,9^, molecular descriptors^10^, and drug associated entities like drug-target^11,12^, drug-gene^13^, and drug-pathway^14^. These data are structured and concise, facilitating effective representation learning and modeling. In contrast, side effect representation learning has seen limited progress, primarily due to its reliance on unstructured, ambiguous, and noisy natural language texts, such as clinical reports, spontaneous narratives, and regulatory documents. Current approaches to side effect representation are mainly categorized into three types: Word Embedding methods^15–17^ using word co-occurrence statistics, ontology-based^18,19^ methods encoding standardized structure from MedDRA^20^ or ADReCS^21^, and knowledge graph methods^22,23^ relying on extracted entity-relations. Characterized by statistic-driven or predefined encoders independent of specific tasks, these representation learning methods struggle in the context of side effect texts, which are mostly symptom-oriented, and stem from diverse causes. Statistic-driven representations overemphasize frequent symptom descriptors, obscuring underlying pharmacological patterns. Predefined structures are static and cannot selectively filter information to accommodate the diverse mechanisms causing side effects. Consequently, side effects caused by common drugs may not be mapped close together in the embedding space, limiting the discriminability of the learned representations and their usefulness for downstream prediction tasks.

Prompt-based LLMs, such as GPT^24^ and Qwen^25^, with their exceptional capabilities in natural language distillation, reasoning, and summarization, offer a transformative opportunity to address this gap^26,27^. Recent studies have leveraged LLMs to extract side effect information from unstructured textual sources, including drug labels^28^ and social media content^29^, as well as to predict drug-drug interactions leading to adverse drug reactions^30,31^. These applications highlight the potential of LLMs to analyze textual data for side effect representations. However, relying on standalone LLMs for end-to-end side effect prediction is inherently inaccurate: Their overconfidence and hallucinations produce erroneous predictions^32^, token-length constraints limit processing of large-scale datasets, and exclusive reliance on textual data prevents leveraging deep learning and multi-modal fusion techniques proven valuable in prior work.

To address representation limitations of predefined encoders and suboptimal prediction of standalone LLM inference, we propose prompt-based side effect prediction (PromptSE), a hybrid framework that combines the semantic reasoning power of LLMs with the predictive precision of deep learning. Unlike prevalent approaches that use LLMs as static encoders or simple feature extractors, PromptSE treats LLMs as dynamic reasoning agents explicitly tailored to the symptom-oriented and heterogeneous nature of side effect narratives. We enhance drug-side effect prediction by first improving side effect profiles via a multi-stage, multi-perspective LLM-reasoning process that distills mechanism-relevant information while suppressing irrelevant symptoms and off-target causes. The resulting profiles are encoded with BioBERT ^33^ and used in a deep learning module for drug-side effect prediction. Building on this framework, PromptSE+ extends the prediction module by integrating multi-modal drug information for enhanced accuracy, demonstrating the framework’s flexibility and compatibility with advanced models. A Hierarchical Graph Convolutional Network (HiGCN) further refines entity representations through controlled message passing, enabling low-frequency entities to benefit from related high-frequency information and thus mitigating weak supervision.

In summary, we improve drug-side effect prediction by enhancing the quality of side effect representations. Our contributions include side-effect focus addressing underexplored challenge, hybrid LLM-deep learning augmenting models, LLM-derived representation uncovering mechanism, and HiGCN refining rare entities.

## Results

We evaluated and visualized our contributions to both side effect representation effectiveness and drug-side effect prediction accuracy. Representation effectiveness is assessed via Kolmogorov-Smirnov (KS) tests^34^ on cumulative similarity distributions of related/unrelated side effect pairs and case studies. For measuring prediction performance on our imbalanced datasets, we chose metrics sensitive to skewed class distributions: area under the precision-recall curve (AUPR), area under the receiver operating characteristic curve (AUC), Matthews correlation coefficient (MCC), and Macro-averaged F1 score (Macro-F1). The closer the metrics are to 1, the better the performance. Detailed formulas of mentioned metrics are provided in the Supplementary Document.

### Evaluation of side effect representations

**Fig. 1:**
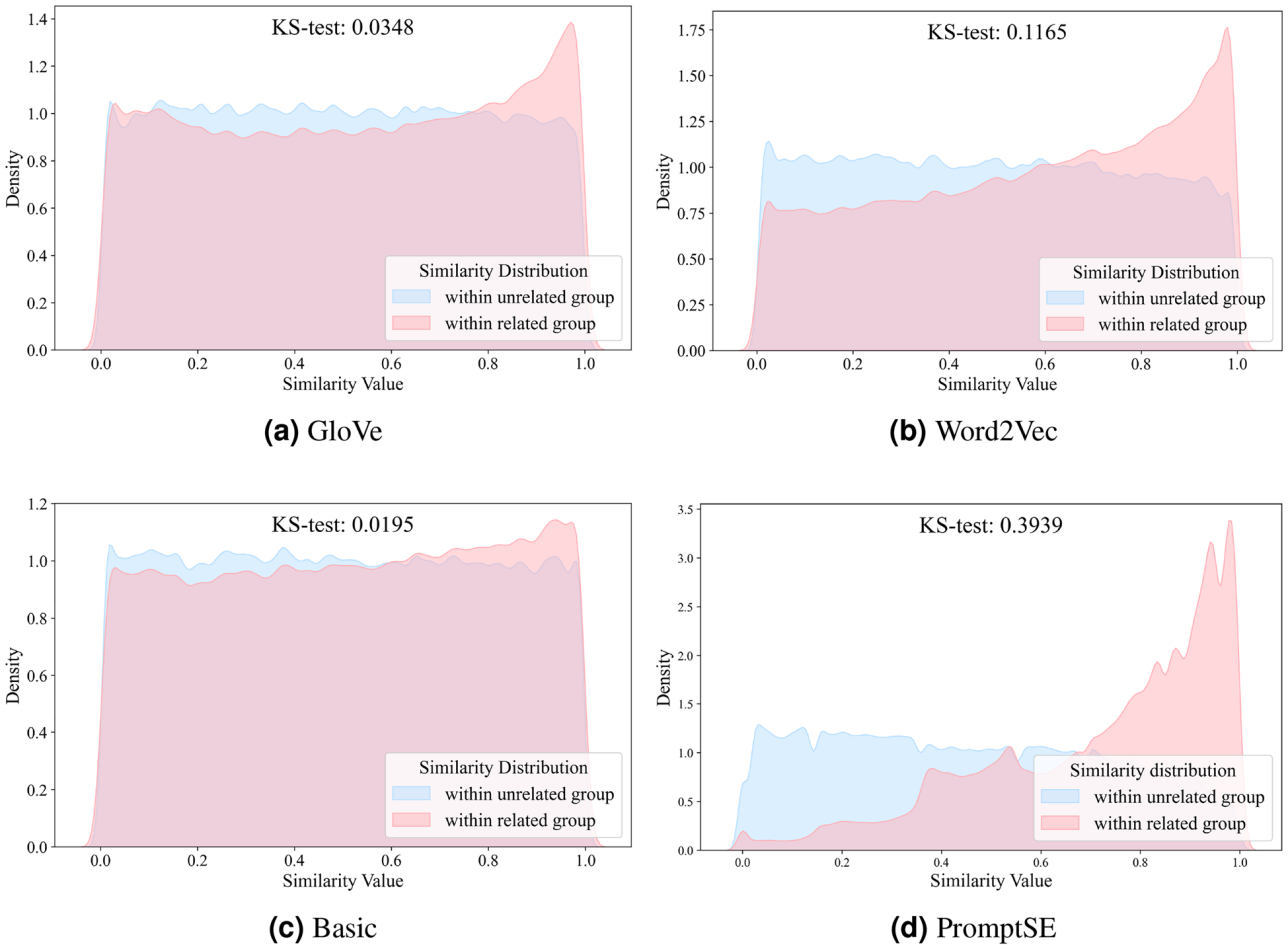
KDE plots of similarity scores for related (pink) and unrelated (blue) side effect pairs across four representation methods: (**a**) GloVe, (**b**) Word2Vec, (**c**) Basic, and (**d**) PromptSE. Each subfigure shows the distribution overlap and KS test statistic, with higher KS values indicating better separation.

We posited that ideal side effect representations should cluster embeddings of side effects caused by common drugs while separating those caused by distinct drugs. To test this hypothesis, we compute the dot product between pairs of side effect embeddings to obtain similarity scores. These pairwise similarities are divided into two groups: the related group, comprising similarities between side effects sharing common causative drugs, and the unrelated group, comprising similarities between side effects caused by different drugs. Similarity scores are quantile-mapped to a uniform distribution between 0 and 1 and visualized using kernel density estimation (KDE) plots, shown as pink and blue shaded areas for related and unrelated groups, respectively. The separation between distributions is quantified using the KS test, as depicted in Fig. 1. To assess the discriminative power of our LLM-derived side effect representations, we compare four types of representations:

- GloVe: 300-dimensional vectors encoding side effect terms using GloVe pretrained on Wikipedia ^16^.
- Word2Vec: 200-dimensional vectors encoding side effect terms using Word2Vec pretrained on a combined PubMed, PMC, and Wikipedia corpus ^15^.
- Basic: 768-dimensional vectors encoding basic descriptions of side effects using pretrained BioBERT. These descriptions, denoted as $\{\alpha _i\}$, are detailed in “Entity characterization” Section.
- PromptSE: 768-dimensional vectors encoding our LLM-derived profiles of side effects using pretrained BioBERT.

Figure 1 shows that PromptSE achieves the highest KS value (0.3939), outperforming GloVe (0.0348), Word2Vec (0.1165), and Basic (0.0195). Among the baselines, GloVe and Word2Vec are widely used word embedding methods, characterized as statistics-driven and predefined term encoders. While Word2Vec benefits from a larger pretraining corpus, both suffer from the symptom-oriented nature of side effect texts. This leads to many related pairs having low similarity, reflected in the pink shaded area staying relatively high on the left side of the x-axis. The Basic one encodes LLM-generated side effects’ descriptions aligning with their MeSH tree numbers, which providing an ontological structure. As shown in Fig. 1c, the pink and blue shaded areas almost overlap, revealing that simply feeding a term to an LLM with a conventional prompt does not produce pharmacologically meaningful profiles. Our PromptSE builds on Basic but uses a multi-stage, multi-perspective prompting tailored to the characteristics of side effect texts, explicitly leveraging the LLM’s reasoning ability to extract underlying pharmacological insights. In Fig. 1d, the pink shaded area for related pairs shifts to the right and separates from the blue one, indicating a superior discriminative ability of our LLM-derived side effect representations.

### Performance comparison with other methods

To comprehensively evaluate the performance of PromptSE and PromptSE+, we conducted comparisons with several representative baselines under both warm-start and cold-start scenarios, with results reported separately. The labeled drug-side effect pairs are divided into a training set $S_{\text {Train}}$ and a test set $S_{\text {Test}}$. In the warm-start setting, the split is performed at the drug-side effect pair level, where labeled pairs are randomly assigned to $S_{\text {Train}}$ and $S_{\text {Test}}$ with a ratio of 9:1, and 5% of $S_{\text {Train}}$ is further used as the validation set. In the cold-start setting, the split is performed at the drug level to evaluate generalization to unseen drugs: 90% of drugs and their associated drug-side effect pairs form $S_{\text {Train}}$, while the remaining 10% of drugs and their associated pairs form $S_{\text {Test}}$.

*Warm-start comparison.* In the warm-start setting, the compared baselines are listed below, and the corresponding results are reported in Table 1.

- MNMF^35^: MNMF reconstructs the drug-side effect adjacency matrix utilizing the nonnegative matrix factorization (NMF) and employs heat diffusion based on the matrix scores to predict drug-side effect associations.
- MSVD: MSVD is a variant of MNMF, replacing NMF with TruncatedSVD for matrix reconstruction.
- idse-HE^36^:idse-HE employs a hybrid graph neural network to fuse drug molecular structures, substructure sequences, and biological network topology, then predicts side effects through adjacency matrix reconstruction.
- DSGAT^37^: DSGAT utilizes graph attention networks with drug molecular graphs and side effect similarity graphs to predict the drug-side effect frequencies. It sets 0.91 as the threshold between unknown associations (0) and frequency class 1. For drug-side effect association prediction, we adopted their suggested threshold of 0.91 to distinguish existing associations.
- MSDSE^10^: MSDSE employs a deep learning framework that integrates multi-scale drug-intrinsic features, including SMILES sequence-based word embedding, substructure-based molecular fingerprint, and chemical structure based graph embeddings, to predict drug-side effect associations.

Our evaluation categorized models into two groups: those without and those with drug information. In the former group, MNMF and MSVD primarily rely on association matrices derived from known drug-side effect associations. Our PromptSE method, however, leverages textual side effect representations, outperforming MSVD by 9.26% on AUPR . Though using only side effect data is impractical for drug discovery, our PromptSE method’s textual side effect representations markedly improve prediction performance, demonstrating their informativeness and utility. For models incorporating drug information, DSGAT, idse-HE, and MSDSE incorporate drug-specific details but lack side effect information. Our PromptSE+ integrates both drug and side effect data, achieving the best performance, surpassing the runner-up (MSDSE) by 1.81% on AUPR . A paired bootstrap significance test with 1000 resamples further confirmed that the AUPR improvement of PromptSE+ over MSDSE is stable rather than due to sampling variability: the mean difference was 0.012, with a 95% confidence interval of [0.008, 0.013], which excludes 0. This improvement demonstrates that PromptSE+ ensures compatibility with state-of-the-art drug-centric methods and enhances prediction accuracy by integrating informative side effect representations.

**Table 1:** Performance comparison under the warm-start setting: Bold indicates the best result without drug information; bold and underlined indicates the best result with drug information.

	Model	AUPR	AUC	Macro-F1	MCC
w/o drug information	MNMF	0.5854	0.9439	0.6159	0.4608
	MSVD	0.5996	0.9469	0.6658	0.4640
	PromptSE	**0.6551**	**0.9603**	**0.7507**	**0.5404**
w/ drug information	DSGAT	0.5170	0.9508	0.7401	0.5043
	idse-HE	0.6547	0.9564	0.7711	0.5765
	MSDSE	0.6756	0.9522	0.7973	0.6070
	PromptSE+	**0.6878**	**0.9628**	**0.7988**	**0.6089**

*Cold-start comparison.* To evaluate generalization to unseen drugs, we further considered a cold-start setting. Under this setting, MNMF, MSVD, and idse-HE were not included because they are designed for transductive learning and are not suitable for this evaluation. We thus compared DSGAT, MSDSE, PromptSE, and PromptSE+, with results reported in Table 2.

PromptSE+ achieves the best overall performance, ranking first on all four metrics. Compared with the strongest baseline MSDSE, PromptSE+ improves AUPR from 0.3408 to 0.3839 . PromptSE also performs strongly, achieving the second-best results on AUC, Macro-F1, and MCC, and surpassing MSDSE on these three metrics. These results suggest that more informative side effect representations provide richer predictive signals, thereby reducing reliance on seen drug-specific information and improving generalization to unseen drugs.

**Table 2:** Performance comparison under the cold-start setting. **Bold** indicates the second-best result; bold and underlined indicates the best result.

Model	AUPR	AUC	Macro-F1	MCC
DSGAT	0.171	0.8501	0.4546	0.1664
MSDSE	**0.3408**	0.8845	0.6281	0.3387
PromptSE	0.3303	**0.8891**	**0.6834**	**0.3671**
PromptSE+	**0.3839**	**0.8962**	**0.7120**	**0.4295**

### Ablation study

To evaluate the contributions of our proposed textual representations and HiGCN module, we conducted ablation experiments on two models, PromptSE and PromptSE+, by generating two variants for each model. These variants are defined as follows:

- W/O textual representations: Removes the textual representations of side effects.
- W/O HiGCN: Removes the HiGCN module.

Table 3 summarizes the predictive performance of the ablated variants. Removing textual side effect representations leads to a substantially larger performance drop than removing HiGCN, indicating that semantic side effect representations are the dominant source of predictive gain. For PromptSE, removing textual representations causes a marked decline across all metrics because no additional drug information is available; for PromptSE+, the degradation is less pronounced, as drug features can partially compensate. The effect of removing HiGCN is comparatively moderate. HiGCN contributes more to PromptSE than to PromptSE+, suggesting that message passing is more beneficial when direct drug information is limited. In particular, HiGCN enriches low-frequency drug and side effect representations with neighbor information, thereby reinforcing positive supervisory signals for these nodes. This effect is reflected more clearly in AUPR, Macro-F1, and MCC, which place greater emphasis on positive-association identification under class imbalance. By contrast, AUC mainly reflects the global ranking between positive and negative pairs, so the gain brought by HiGCN on AUC is limited and is even slightly unfavorable for PromptSE+.

**Table 3:** Performance of PromptSE and PromptSE+ variants in ablation study.

Model	Variant	AUPR	AUC	Macro-F1	MCC
PromptSE	W/O textual representations	0.5239	0.9358	0.6001	0.3344
	W/O HiGCN	0.6455	0.9594	0.7336	0.5172
PromptSE+	W/O textual representations	0.6813	0.9547	0.8040	0.6151
	W/O HiGCN	0.6861	0.9645	0.7969	0.6063

### Case study

To evaluate the effectiveness of our proposed LLM-derived side effect representations, we conducted two complementary case studies. The first case study examines the mechanism-oriented inference of drug-side effect relationships, and the second case study analyzes the side effect profiles.

Table 4 compares conventional prompting and our stepwise prompting for “Alitretinoin *↔* abdominal distension” and “Pindolol *↔* Somnolence”. Conventional prompting leads to vague responses, such as “no direct evidence”, “not a commonly” and “not fully understood”, without actionable insights. In contrast, our method applies multi-perspective prompting to distill mechanism-relevant information from side effect texts that stem from diverse causes, while suppressing irrelevant symptoms and off-target explanations. The generated text captures pharmacologically relevant cues that are broadly consistent with published pharmacology. For example, in the alitretinoin case, the generated description reflects gastrointestinal, epithelial, and immune-related relevance supported by retinoid receptor biology and reported gastrointestinal adverse effects ^38–40^, while in the Pindolol case it reflects blood-brain barrier penetration together with clinically reported central nervous system effects ^41–43^.

**Table 4:** Comparison of drug-side effect association inferences using conventional prompting versus our stepwise prompting.

Drug *↔* Side Effect	Case	Inferred Drug-Side Effect Relationship
Alitretinoin *↔* Abdominal distension	Conventional prompting	Alitretinoin is a medication used to treat severe hand eczema and other skin diseases, and it is a derivative of vitamin A. Although there is currently **no direct evidence** to indicate that Alitretinoin causes the side effect of abdominal distension.
	Stepwise prompting	Alitretinoin’s distribution includes the **digestive system (epithelial tissues, immune cells)**, where **RXR activation** disrupts gut motility and secretion, leading to gas/fluid accumulation and abdominal distension.
Pindolol *↔* Somnolence	Conventional prompting	Somnolence is** not a commonly** reported side effect of pindolol. However, some patients may experience drowsiness or fatigue. The exact mechanism by which pindolol might cause somnolence is **not fully understood**, but it could be related to its effects on the central nervous system.
	Stepwise prompting	Pindolol’s moderate **lipophilicity** (log P *∼* 2.3) and indole ring system enable **blood-brain barrier penetration**, leading to CNS beta-adrenergic partial agonism and somnolence.

**Fig. 2:**
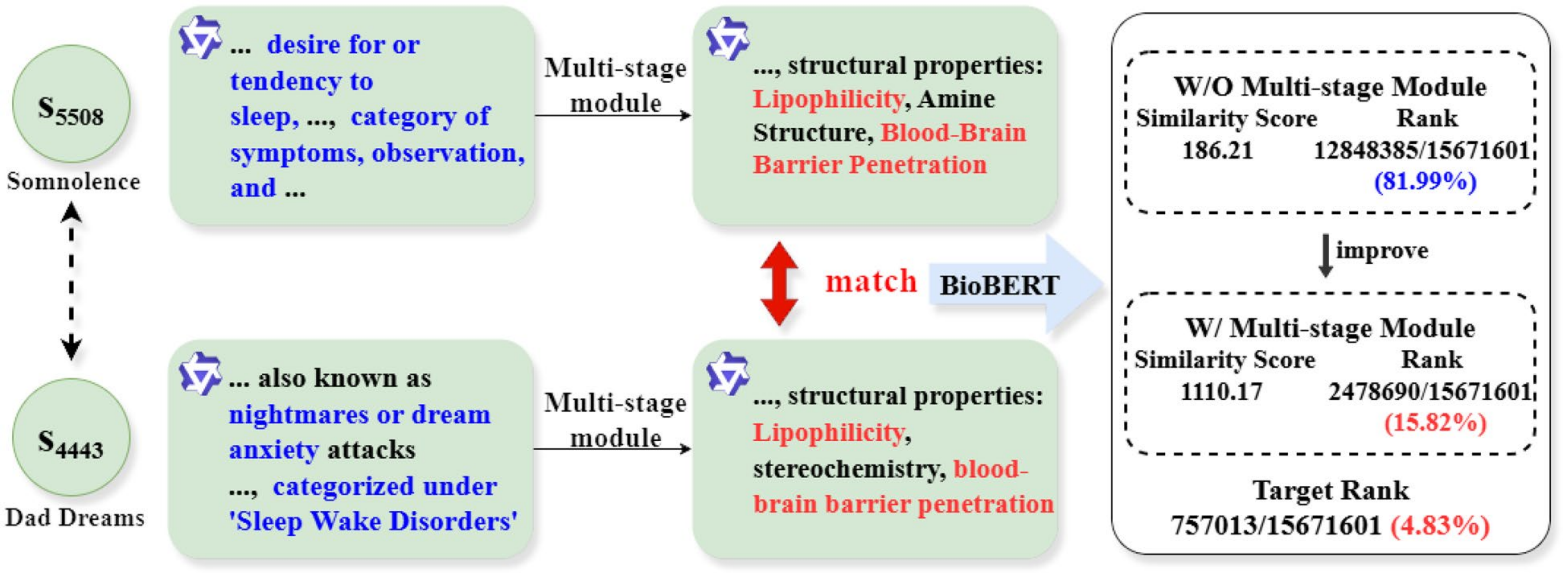
Comparison of side effect similarity captured by basic descriptions versus LLM-derived profiles.

We examined the side effect profiles for “somnolence” and “bad dreams” to evaluate the semantic coherence of our multi-stage, multi-perspective prompting approach. Figure 2 compares basic descriptions $\{\alpha _i\}$ with our LLM-derived profiles. The basic descriptions are largely symptom-oriented, characterizing “somnolence” as “a desire or tendency to sleep” and “bad dreams” as “anxiety attacks”, indicating limited semantic overlap. In contrast, our LLM-derived profiles reveal pharmacologically grounded descriptions: both side effects are characterized by consistent mechanistic terms such as “lipophilic” and “blood-brain barrier penetration”. This demonstrates that our multi-stage approach effectively distills mechanism-relevant information while suppressing irrelevant symptom-level content.

Next, we evaluate representation quality from the perspective of similarity-based ranking. To construct a pharmacologically motivated reference ranking, we first counted the number of drugs shared by each pair of side effects, meaning the number of drugs known to cause both side effects. A larger shared-drug count indicates stronger pharmacological relatedness, so in an ideal embedding space, such pairs should be represented more closely. Sorting all side effect pairs in descending order of shared-drug counts yields a reference ranking in which “somnolence” and “bad dreams” are placed at 757,013/15,671,601 (top 4.83%). We then encoded our LLM-derived profiles using pretrained BioBERT, computed dot product similarities, and ranked all pairs in descending order of similarity. Under this ranking, “somnolence” and “bad dreams” are placed at 2,478,690/15,671,601 (top 15.82%). In comparison, when encoding the basic descriptions with the same BioBERT model and ranking by dot product similarity, the pair is placed at 12,848,385/15,671,601 (top 81.99%). The closer alignment of our LLM-derived ranking to the reference ranking indicates that our representations better capture pharmacological relatedness and provide more pharmacological insights than conventional prompt-generated ones.

### Robustness of LLM-derived side effect representations

**Fig. 3:**
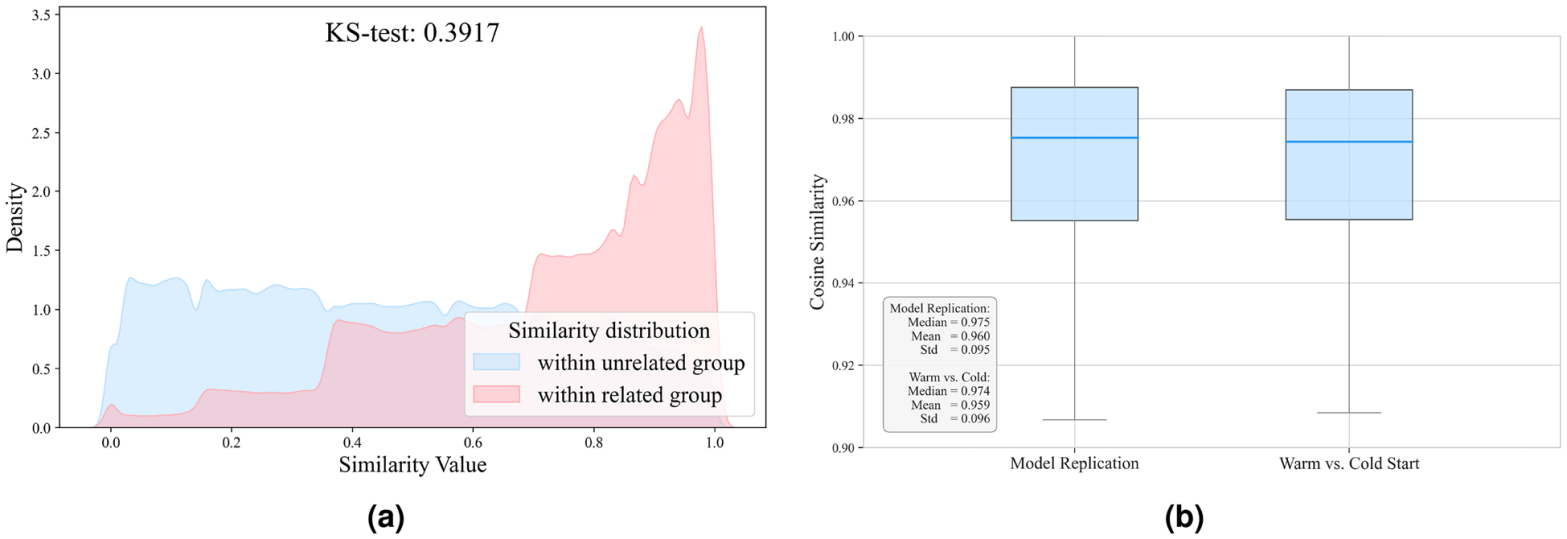
Robustness analysis of LLM-derived side effect representations: (**a**) KDE plots after random projection from 768 to 200 dimensions; (**b**) box plots of cosine similarities from repeated LLM runs and from warm-start versus cold-start settings.

We further evaluated the robustness of LLM-derived side effect representations with respect to both dimensionality reduction and generation stability. To examine whether their strong discriminative power mainly comes from the higher dimensionality, we reduced the PromptSE vectors to 200 dimensions via random projection, matching the dimensionality of Word2Vec. As shown in Fig. 3a, the separation between related and unrelated side effect pairs remains almost unchanged, with the KS statistic only slightly decreasing from 0.3939 to 0.3917. This indicates that the discriminative power of LLM-derived representations are well preserved after dimensionality reduction.

We also assessed the stability of the LLM-derived representations under repeated generation. Specifically, we generated the side effect profiles twice, encoded them with BioBERT, and computed the cosine similarity between the two embeddings for each side effect. As shown in Fig. 3b, the average cosine similarity is 0.975, indicating high stability across repeated runs. This supports our use of a single generated profile for each side effect throughout the experiments. In addition, the average cosine similarity between the representations generated under the warm-start and cold-start settings is 0.974, showing that the exclusion of a subset of drugs in the cold-start setting has little effect on the resulting side effect profiles.

## Discussion

This study presents a novel framework that leverages LLMs to improve side effect representations, thereby enhancing the accuracy of drug-side effect prediction. Existing LLM-based approaches in drug-related predictive tasks typically follow two paths. The first treats LLMs as pretrained encoders or conventional extractors and allows straightforward integration with machine/deep learning models but underutilizing the LLMs’ reasoning power. The second fully exploits LLM reasoning in an end-to-end manner, prompting the model to generate predictions directly from text, without leveraging existing machine/deep learning predictors. Our framework bridges these extremes. It leverages the distillation, reasoning and summarization capabilities of LLMs to derive essential and informative representations. These representations are then used in deep learning predictors, combining reasoning-rich features with the strengths of established models. Experiments show that these representations are more informative than those obtained from conventional prompting, and the resulting predictors achieve state-of-the-art performance on benchmark datasets, suggesting potential advantages over purely end-to-end LLM approaches. Beyond drug-side effect prediction, this framework offers a transferable paradigm for integrating LLM reasoning into predictive modeling, with potential applications in drug-disease association prediction, drug-drug interaction detection, and other tasks requiring reasoning-driven textual representations. In this context, systematic comparison with external datasets, curated pharmacological knowledge bases, and published pharmacological evidence is important for further strengthening the biological grounding and generalizability of LLM-derived representations.

## Methods

### Dataset

This study formulates drug-side effect prediction as a binary classification task, predicting whether a drug-side effect pair is associated (positive) or not (negative) based on their vector representations. The analysis leverages three datasets: a labeled drug-side effect dataset, drug features, and side effect features.

The labeled drug-side effect dataset^35^ combines the DrugBank and SIDER databases through PubChem and DrugBank ID mappings. It captures 133,750 known associations between $N_{\text {Dr}}=1,020$ drugs, denoted as $S_{\text {Dr}} = \{d_1, d_2, \dots , d_{N_{\text {Dr}}} \}$, and $N_{\text {SE}} = 5,599$ side effects, denoted as $S_{\text {SE}} = \{s_1, s_2, \dots , s_{N_{\text {SE}}}\}$. The sample set encompasses 5,710,980 possible drug-side effect pairs from the Cartesian product $S_{\text {Dr}} \times S_{\text {SE}}$. Each pair $(d_i, s_j)$ is assigned a binary label $y_{ij} \in {0, 1}$. Only *2.34%* of samples are positive, indicating significant class imbalance.

Drug features, following MSDSE^10^, include SMILES sequences, substructure-based molecular fingerprints, and chemical structures for $S_{\text {Dr}}$. Side effect features are derived from Medical Subject Headings(MeSH) Tree Numbers in the MetaADEDB database^44^. MeSH Tree Numbers are unique identifiers assigned to side effects within the MeSH hierarchical biomedical taxonomy, representing their structural categorization. A multi-stage LLMs-based method, detailed later, incorporates MeSH Tree Numbers to generate structured side effect profiles.

Drug-side effect associations are represented by an adjacency matrix $A \in \{0, 1\}^{N_{\text {Dr}} \times N_{\text {SE}}}$ where $a_{ij} = y_{ij}$ indicates the presence (1) or absence (0) of an association between drug $d_i$ and side effect $s_j$. From this, we derive two normalized similarity matrices:

1 $$\begin{aligned} A^{\text {Dr}} = {D_{\text {Dr}}}^{-1/2} A {A}^T {D_{\text {Dr}}}^{-1/2} \in \mathbb {R}^{N_{\text {Dr}} \times N_{\text {Dr}}}, \quad A^{\text {SE}} = {D_{\text {SE}}}^{-1/2} {A}^T A {D_{\text {SE}}}^{-1/2} \in \mathbb {R}^{N_{\text {SE}} \times N_{\text {SE}}}, \end{aligned}$$

where $D_{\text {Dr}}$ and $D_{\text {SE}}$ are diagonal degree matrices with the (*i*, *i*)-entries being $\sum _k (A A^T)_{ik}$ and $\sum _k (A^TA)_{jk}$, respectively. These normalized matrices respectively quantify drug and side-effect similarities by counting common associations.

### Overview of PromptSE

**Fig. 4:**
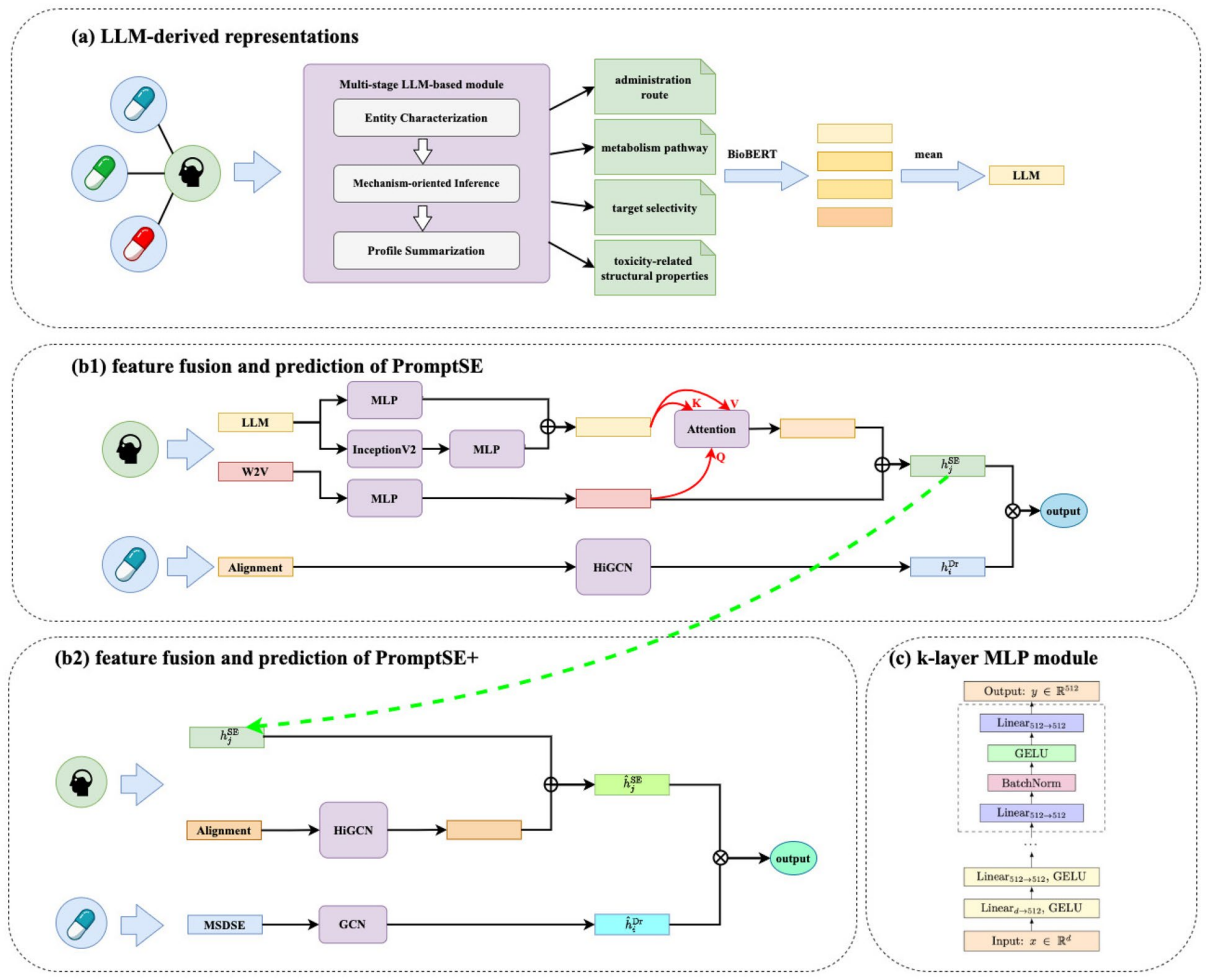
Overview of PromptSE and PromptSE+ Framework. (**a**) LLM-derived representations, where side-effect profiles are generated using stepwise LLM reasoning and embedded with BioBERT. (**b1**) PromptSE’s feature fusion and prediction stage, combining LLM-derived and Word2Vec side-effect features with alignment drug vectors refined by HiGCN. (**b2**) PromptSE+’s feature fusion and prediction stage, replacing alignment drug vectors with advanced drug features from MSDSE for enhanced performance. (**c**) The general *k*-layer MLP architecture used in (**b1**) and (**b2**).

PromptSE leverages textual data and a HiGCN to enhance side effect representations for improved drug-side effect prediction. As illustrated in Fig. 4, PromptSE comprises two primary stages: (a) LLM-derived representations, and (b) feature fusion and prediction, with distinct implementations for PromptSE and PromptSE+ in stage (b), as depicted in parts (b1) and (b2). In stage (a), a three-stage, four-perspective LLM-reasoning process generates side effect profiles using side effect terms, MeSH Tree Numbers, and partial drug-side effect associations, which are embedded via BioBERT into computational pharmacological vectors. In stage (b), PromptSE (b1) combines two types of side effect textual features: LLM-derived representations and Word2Vec-generated vectors^15^. These features are fused using multi-layer perceptrons, an inception module, and a multi-head attention neural network to produce comprehensive side effect representations. Drug representations are learnable alignment vectors refined by our proposed HiGCN. The association probability between drugs and side effects is computed as the dot product of their respective embeddings. PromptSE+(b2) extends this framework by incorporating state-of-the-art drug features, such as those from MSDSE, achieving superior performance over the state-of-the-art models. The following sections elaborate on PromptSE and PromptSE+.

### LLM-derived representations

The unstructured, symptom-oriented, and heterogeneous nature of side effect data, derived from sources like clinical reports and spontaneous reporting systems, poses significant challenges for representation learning, such that current methods struggle to capture pharmacologically relevant patterns. For example, side effects like “acute abdomen” and “abdominal cramps”, both potentially linked to NSAIDs, are described with distinct clinical terminology. “Acute abdomen” highlights sudden onset and severity, whereas “Abdominal cramps” emphasizes muscular contractions. This textual variability leads classifiers to prioritize superficial linguistic features over mechanistic relationships, degrading prediction accuracy.

To address this, we propose a three-stage, four-perspective LLM-reasoning approach to generate pharmacologically meaningful side effect profiles from known drug-side effect associations. The indirect nature of side effects, which are not intended outcomes of a drug’s therapeutic effects, complicates LLM modeling without domain-specific guidance. As illustrated in “Case study” section, LLMs often respond with generic answers, such as “no direct evidence” or “not fully understood”. Inspired by chain-of-thought reasoning^45^, our multi-stage prompting guides LLMs to construct discriminative representations of side effects through three structured stages: entity characterization, mechanism-oriented inference, and profile summarization. To mitigate LLMs’ tendency to produce generic responses, we constrain the analysis to four key pharmacological perspectives known to influence side effects: administration route, metabolism pathway, target selectivity, and structural properties. Using Qwen3-Plus-2025-12-01, an API-accessible large language model, this framework captures mechanistic drug-side effect relationships. The following sections detail each stage of the framework.

#### Entity characterization

Entity characterization serves as the foundational stage of our stepwise LLM-reasoning module. This stage acquires comprehensive and standardized descriptions for drugs and side effects, denoted as $\alpha _j$ for each side effect $s_j \in S_{\text {SE}}$ and $\beta _i$ for each drug $d_i \in S_D$. These descriptions underpin subsequent mechanism-oriented inference and profile summarization.

For side effects, we employ Qwen to produce descriptions aligned with MeSH Tree Numbers, ensuring normative statements to support downstream analyses. The corresponding prompt is provided in the Supplementary Document (Prompt A). For drugs, we use a two-step process to derive $\beta _i$. First, we identify target information $t_i$ for each drug $d_i$. Then, using the pair $(d_i, t_i)$, Qwen infers $\beta _i$, capturing key properties such as routes of administration, anatomical location of action, target distribution, structural or toxicological features, and metabolic pathways. These attributes are critical for side effect prediction. Prompts for acquiring $t_i$ and inferring $\beta _i$ are included in the Supplementary Document (Prompt B and C).

#### Mechanism-oriented inference

To perform mechanism-oriented inference for drug-side effect associations using an LLM, we define the index set $I^{\text {SE}}_j = \{ i:(d_i, s_j)\in S_{\text {train}} \ \text {and} \ y_{ij} = 1 \}$ for the *j*-th side effect, representing indices of all the drugs associated with $s_j$ in the training set only, thereby avoiding information leakage from the test set. To mitigate the computational cost and avoid overfitting for high-frequency side effects, we randomly sample a subset of $I^{\text {SE}}_j$ denoted as $\tilde{I}_j$. Specifically,

$$\begin{aligned} \tilde{I}_j = {\left\{ \begin{array}{ll} \{ i_1, \cdots , i_{20}\} \subset I^{\text {SE}}_j & |I^{\text {SE}}_j| \ge 20, \\ I^{\text {SE}}_j & \text {otherwise}. \end{array}\right. } \end{aligned}$$

For each side effect $s_j$ and drug $d_i$ with $i \in \tilde{I}_j$, the LLM leverages descriptions $\beta _i$ and $\alpha _j$ to infer causality, denoted as $c_{ij}= f(d_i, \beta _i, s_j, \alpha _j)$. The inference $c_{ij}$ identifies the causal category-administration route, metabolism pathway, target selectivity, or structural properties-along with the specific cause and the LLM’s reasoning. For administration route, it details the route and affected organs or systems; for metabolism pathway, the metabolizing organ and substances; for target selectivity, the target distribution location; and for structural properties, the precise structure and characteristics. This approach ensures systematic and interpretable causal analysis for drug-side effect interactions. Detailed prompts refer to the Supplementary Document (Prompt D).

#### Profile summarization

For each side effect $s_j$, we construct a profile summarizing drug-induced side effect causes across the four mentioned categories, denoted $p^{(1)}_j, p^{(2)}_j, p^{(3)}_j, p^{(4)}_j$. These summaries are derived via

$$\begin{aligned} p_j := (p^{(1)}_j, p^{(2)}_j, p^{(3)}_j, p^{(4)}_j) = g(s_j, \alpha _j, {c_{ij}: i\in \tilde{I}_j}), \end{aligned}$$

using mechanism-oriented inferences $\{c_{ij}: i \in \tilde{I}_j\}$, side effect description $\alpha _j$, and side effect $s_j$. If no causal factor is identified for a given category, the corresponding summary is assigned as “None”. Detailed prompts are provided in the yy Document (Prompt E).

To generate a computational representation, each textual summary $p^{(k)}_j$ is transformed into a 768-dimensional vector $h(p^{(k)}_j)$. If $p^{(k)}_j$ is “None”, it is mapped to a 768-dimensional zero vector; otherwise, it is encoded as ${\textbf {bert}}(p^{(k)}_j)$ using BioBERT. The final representation $\ell _j$ is the average of the four category-specific vectors:

2 $$\begin{aligned} \ell _j = \frac{1}{4}\sum _{k=1}^4 h(p^{(k)}_j), \quad h(p) = {\left\{ \begin{array}{ll} {\textbf {0}} & \text {p is None}, \\ {\textbf {bert}}(p) & \text {otherwise}. \end{array}\right. } \end{aligned}$$

The collection $L = [\ell _1, \ell _2, \cdots , \ell _{N_{\text {SE}}}]^T \in \mathbb {R}^{5599*768}$, encapsulates the robust LLM-derived representations of side effects.

### Feature fusion and prediction

#### Side effect feature fusion

This section describes the fusion of side effect representations *L*, defined in Eq. (2), with Word2Vec-derived word embeddings *W*, defined in Eq. (3) to create comprehensive side effect features using Multi-layer Perceptrons (MLPs), inception modules, and attention mechanisms. The *k*-layer MLP, denoted by $\text {MLP}^{k}_{d\rightarrow 512}$, transforms a *d*-dimensional input into a 512-dimensional output, as shown in Fig. 4c. Following the configuration of MSDSE, we use GELU as the activation function in this MLP, where the Gaussian Error Linear Unit (GELU) is defined as $\textrm{GELU}(x)=x\Phi (x)$ and $\Phi (\cdot )$ denotes the cumulative distribution function of the standard Gaussian distribution. For *k\ge 2*, it is defined as:

$$\begin{aligned} \text {MLP}^{k}_{d\rightarrow 512}: {\left\{ \begin{array}{ll} h_i = \text {GELU}(\Theta _i h_{i-1} + b_i), \ \forall i=1,\cdots ,k-2, \\ h_{k-1} = \text {GELU}(\text {BN}(\Theta _{k-1} h_{k-2} + b_{k-1})), \\ y = \Theta _k h_{k-1} + b_k, \end{array}\right. } \end{aligned}$$

where $h_0 \in \mathbb {R}^d$ is the input, $y \in \mathbb {R}^{512}$ is the output, the weights $\Theta _1 \in \mathbb {R}^{512 \times d}$, $\Theta _i \in \mathbb {R}^{512 \times 512}$ for *i=2,… ,k*, and $b_i \in \mathbb {R}^{512}$.

Feature fusion begins by processing *L* through two parallel branches. First, a 4-layer MLP transforms each $\ell _i$, yielding:

$$\begin{aligned} H_L^{\text {MLP}} = [\text {MLP}_{768\rightarrow 512}^{4}(\ell _1), \text {MLP}_{768\rightarrow 512}^{4}(\ell _2), \cdots , \text {MLP}_{768\rightarrow 512}^{4}(\ell _{N_{\text {SE}}})]^T \in \mathbb {R}^{5599 \times 512}, \end{aligned}$$

capturing primary patterns. Second, an adapted Inception-v2 module^46^ using 1D convolutions for sequential embeddings is employed to capture the local structural pattern of LLM-derived representations. Each convolutional block, denoted by $\text {Conv}_{c_i \rightarrow c_o}^k$, consists of an independent single-layer 1D CNN (with input channels $c_i$, output channels $c_o$, and kernel size *k*), followed by batch normalization and the GELU activation function, consistent with the configuration of MSDSE. The average pooling layer with kernel size 3 is denoted by $\text {AvgPooling}^3$. Given *H* as input, the output of the Inception module is formulated as below:

$$\begin{aligned}&C_1 = \text {Conv}_{768\rightarrow 128}^1 (L) \in \mathbb {R}^{5599 \times 128} \\&C_2 = \text {Conv}_{128\rightarrow 128}^3\big ( \text {Conv}_{768\rightarrow 128}^1 (L) \big ) \in \mathbb {R}^{5599 \times 128} \\&C_3 = \text {Conv}_{128\rightarrow 128}^3\Big ( \text {Conv}_{128\rightarrow 128}^3\big ( \text {Conv}_{768\rightarrow 128}^1 (L) \big ) \Big ) \in \mathbb {R}^{5599 \times 128} \\&C_4 = \text {Conv}_{768\rightarrow 128}^1\big ( \text {AvgPooling}^3 (L) \big ) \in \mathbb {R}^{5599 \times 128} \\&H_{\text {CON}} = [C_1, C_2, C_3, C_4] \in \mathbb {R}^{5599 \times 512}. \end{aligned}$$

This is processed by a 2-layer MLP, yielding $H_L^{\text {Inc}} = \text {MLP}_{512\rightarrow 512}^{2}(\text {Inception}(H_{\text {CON}}))$. Having extracted features from both branches, we combine them to a unified representation with a learnable weight $\lambda _1$: $H_{\text {LLM}} = H^{\text {MLP}}_L + \lambda _1 H_L^{\text {Inc}}$.

For word embeddings, each side effect $s_j$ is represented as a 200-dimensional vector $\omega _j$ using Word2Vec, forming

3 $$\begin{aligned} W = [\omega _1, \omega _2, \dots , \omega _{N_{\text {SE}}}]^T \in \mathbb {R}^{5599 \times 200}. \end{aligned}$$

A 3-layer MLP processes the *W* and obtain $H_{\text {W2V}} = \text {MLP}_{200\rightarrow 512}^{3}(W) \in \mathbb {R}^{5599 \times 512}$.

To integrate $H_{\text {LLM}}$ and $H_{\text {W2V}}$, a two-head self-attention mechanism is used. Formally, each head *h* computes:

$$\begin{aligned} \text {Attn}_h(X_1, X_2) = \text {softmax}\left( \frac{(X_1\Theta _h^Q)(X_2\Theta _h^K)^T}{\sqrt{512}} \right) (X_2\Theta _h^V), \end{aligned}$$

where $\Theta _h^Q, \Theta _h^K, \Theta _h^V \in \mathbb {R}^{512 \times 512}$ are head-specific projections and $X_1, X_2$ are input representations. The integrated outputs are concatenated and transformed as:

$$\begin{aligned} H_{\text {attn}} = [\text {Attn}_1(H_{\text {LLM}}, H_{\text {W2V}}), \text {Attn}_2(H_{\text {LLM}}, H_{\text {W2V}})] \Theta ^f, \end{aligned}$$

The final representation combines $H_{\text {W2V}}$ and $H_{\text {attn}}$ with a learnable weight $\lambda _2$:

4 $$\begin{aligned} H^{\text {SE}} = H_{\text {W2V}} + \lambda _2 \text {MLP}(H_{\text {attn}}), \end{aligned}$$

yielding refined features that preserves cross-modal relationships of side effects.

**Fig. 5:**
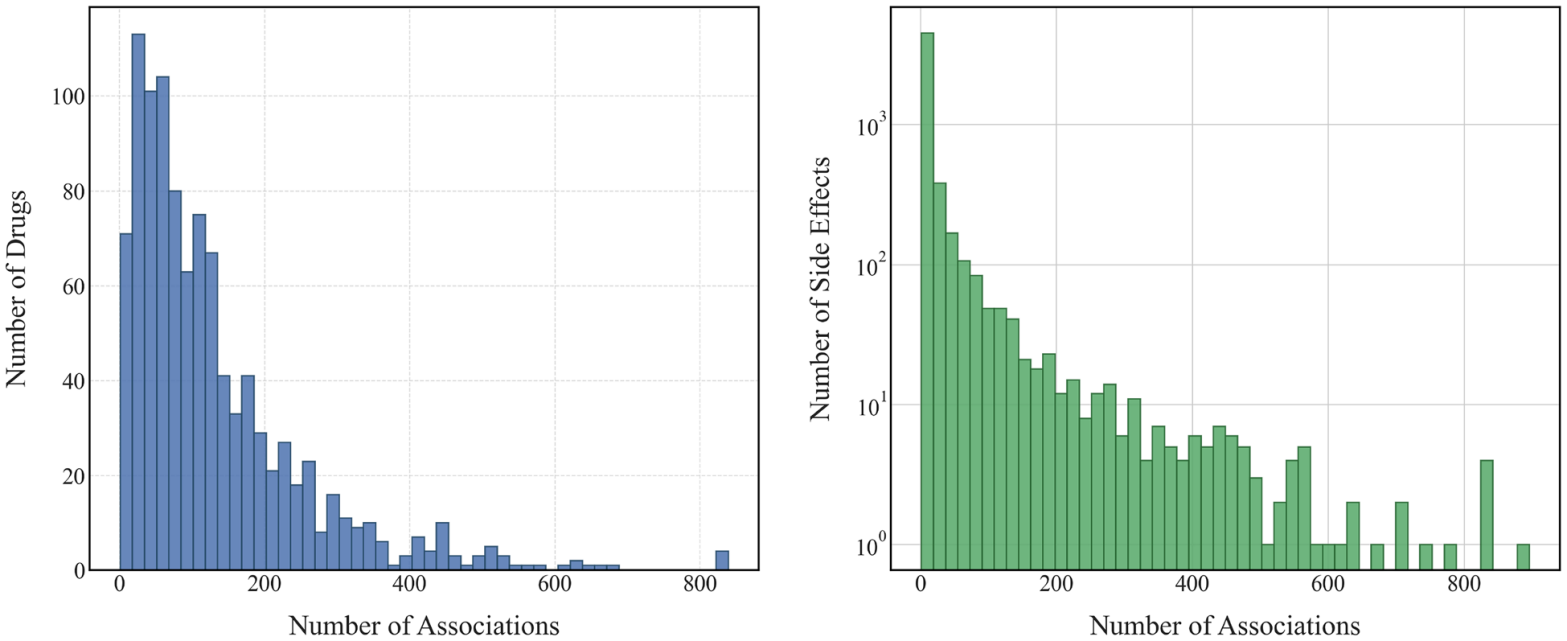
Distribution of associations per drug (left panel) and side effect (right panel).

#### Drug feature as HiGCN-based alignment

In scenarios where drug-specific properties are unavailable, drug representations are initialized as standardized random vectors $H_{\text {rand}}^{\text {Dr}}$ (mean = 0, standard deviation = 0.1). These embeddings are trained using known drug-side effect associations (positive labels) to align with $H^{\text {SE}}$ defined in Eq. (4). However, the highly skewed distribution of these associations (see Fig. 5), with most drugs having few known links, limits training signals for low-frequency drugs, resulting in poor feature updates and suboptimal representations.

Graph convolutional network (GCN)^47^ offers a potential solution to this issue by leveraging message passing to enrich low-frequency drug representations with neighbor information. From a geometric viewpoint, this can be understood as exploiting the local neighborhood structure of multiple latent drug groups, where nearby drugs in the graph are expected to have locally smooth representations. Message passing can therefore partially compensate for limited supervision on sparse nodes. However, standard GCN propagation is bidirectional and does not explicitly constrain cross-group information flow, thereby allowing information to flow from high-frequency to low-frequency drugs and vice versa. This may inadvertently distort well-trained representations of high-frequency drugs. As highlighted by Feng et al.^48^ and Xie et al.^49^, aggregation over graph neighborhoods can also propagate noise from irrelevant or noisy nodes, ultimately degrading target node representations.

To alleviate this problem, we propose the Hierarchical Graph Convolutional Network (HiGCN), which controls the direction of information flow. In this way, HiGCN retains the benefit of local geometric smoothing for low-frequency drugs while reducing the risk of degrading already reliable representations.

**Fig. 6:**
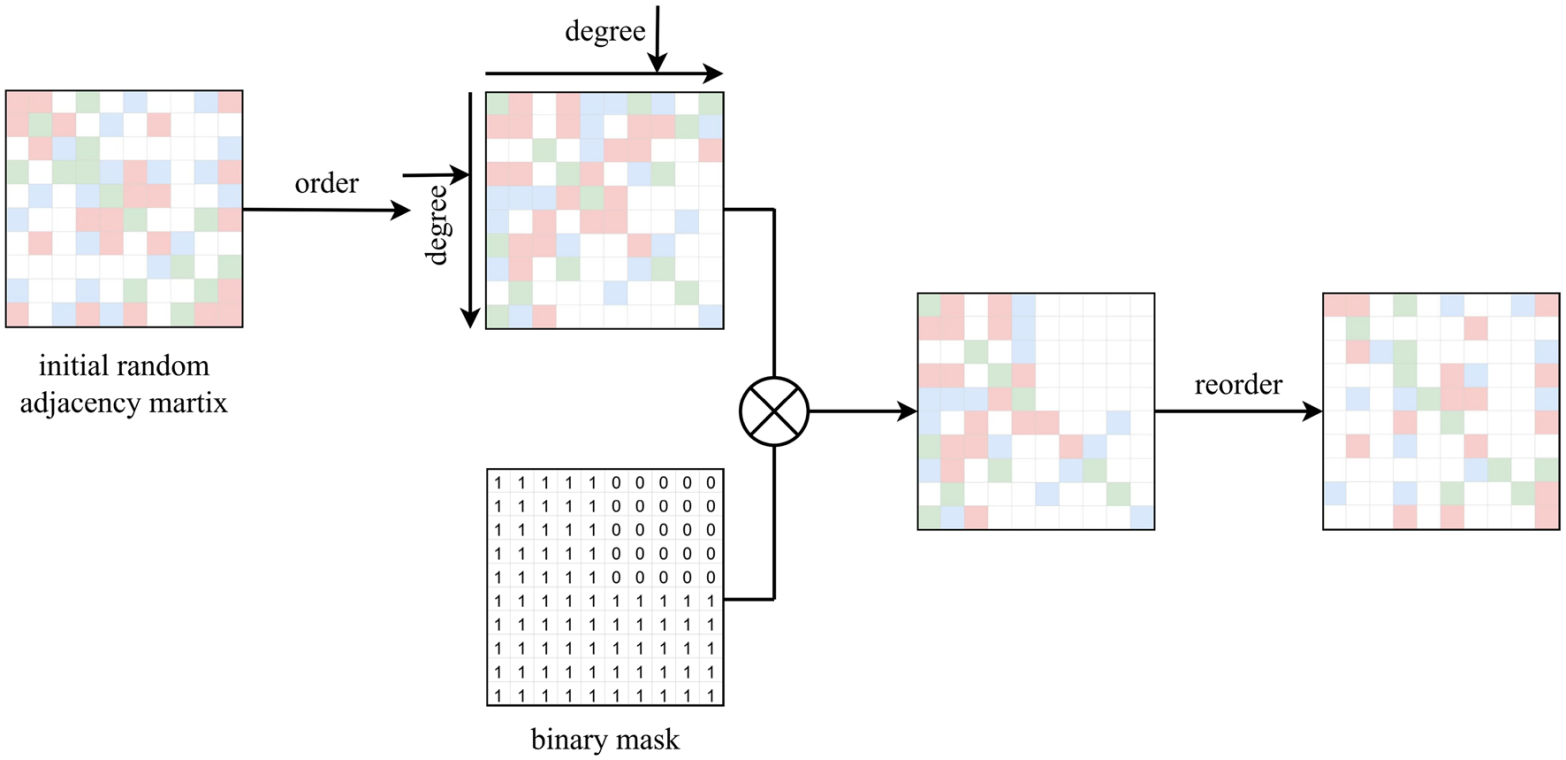
Hierarchical adjacency matrix construction for HiGCN (*K=2*).

HiGCN constructs modified adjacency matrices to enable hierarchical message passing. We begin by defining the degree for drugs and side effects as:

$$\begin{aligned} \text {degree}(d_i) = \sum _{j=1}^{N_{\text {Dr}}} \textbf{1}_{x>0}(a^{Dr}_{ij}), \quad \text {degree}(s_j) = \sum _{i=1}^{N_{\text {SE}}} \textbf{1}_{x>0}(a^{\text {SE}}_{ij}), \end{aligned}$$

where $a^{\text {Dr}}_{ij}$ and $a^{\text {SE}}_{ij}$ are the (*i*, *j*)-entries of the drug-drug ($A^{\text {Dr}}$) and side effect-side effect ($A^{\text {SE}}$) adjacency matrices, respectively. $\textbf{1}_{x>0}(a)$ is an indicator function that returns 1 if *a>0* and 0 otherwise. To establish a hierarchical structure, drug indices are partitioned into *K* ordered tiers, $\cup _{k=1}^K J^{\text {Dr}}_k = \{1, \cdots , N_{\text {Dr}}\}$, such that drugs in a preceding tier have degrees no less than those in a succeeding tier. Specifically, we reorder the drug indices in descending degree $J = (i_1, i_2, \cdots , i_{N_{\text {Dr}}})$, satisfying $\text {degree}(d_{i_1}) \ge \text {degree}(d_{i_2}) \ge \cdots \ge \text {degree}(d_{i_{N_{\text {Dr}}}})$. The drug indices are grouped into *K* tiers:

$$\begin{aligned} J^{\text {Dr}}_k = {\left\{ \begin{array}{ll} \{ i_{(k-1) \lceil \frac{m}{K} \rceil +1}, \cdots , i_{k \lceil \frac{m}{K} \rceil }\} & k = 1, \cdots , K-1, \\ \{i_{(k-1) \lceil \frac{m}{K} \rceil +1}, \cdots , i_m\} & k = K, \end{array}\right. } \end{aligned}$$

where $\lceil \cdot \rceil$ denotes the ceiling function. A modified adjacency matrix $\tilde{A}^{\text {Dr}} = \big ( \tilde{a}^{\text {Dr}}_{ij} \big )$ is constructed from $A^{\text {Dr}}=\big (a^{\text {Dr}}_{ij} \big )$ and *K* as:

$$\begin{aligned} \tilde{a}^{\text {Dr}}_{ij} = {\left\{ \begin{array}{ll} a_{ij}^{\text {Dr}} & i \in J^{\text {Dr}}_s, j \in J^{\text {Dr}}_t, s \ge t, \\ 0 & \text {otherwise}. \end{array}\right. } \end{aligned}$$

Figure 6 illustrates our hierarchical adjacency matrix construction for *K=2* tiers. Initially, we order the initial random adjacency matrix by the descending degrees of drugs, placing high-frequency entities in the top-left and low-frequency ones in the bottom-right. A binary mask then sets the top-right block to zero. These removed connections originate from low-frequency entries but target high-frequency ones. By eliminating these specific connections, we prevent potentially misleading signals from well-represented nodes affecting less reliably represented low-frequency nodes, thus preserving high-frequency representation quality. The matrix is then reordered back to its original indexing, forming the $\tilde{A}$ used in HiGCN.

The HiGCN layer updates embeddings as:

5 $$\begin{aligned} H^{\text {Dr}} = \text {HiGCN} \big (A^{\text {Dr}}, H_{\text {rand}}^{\text {Dr}}, K, \lambda _3 \big ) = H_{\text {rand}} + \lambda _3 \text {ReLU} \big (\tilde{D}^{-\frac{1}{2}} \tilde{A}^{\text {Dr}} \tilde{D}^{-\frac{1}{2}}H_{\text {rand}}^{\text {Dr}} \Theta ^{hi} \big ), \end{aligned}$$

where $\lambda _3$ is a learnable parameter, $\tilde{D}$ is the diagonal degree matrix of $\tilde{A}^{\text {Dr}}$, and $\Theta ^{hi}$ is the learnable weight matrix. ReLU is used in HiGCN following the common practice in GCN. *K* represents how finely we differentiate hierarchical levels and we set *K=2* for PromptSE.

#### Enhanced PromptSE+ extension

Building upon the strong foundation of existing works that excel in drug feature extraction, we further enhance our model’s capabilities by integrating these state-of-the-art drug feature vectors as a valuable complement to our novel side effect representation framework.

Specifically, for drug features, we leverage the state-of-the-art representations $H_{\text {MSDSE}}$ from the MSDSE work^10^. These features are meticulously constructed from various structural features, including fingerprints, SMILES strings, and molecular descriptors. A GCN then processes this initial representation to obtain the final drug embedding, denoted as

6 $$\begin{aligned} \hat{H}^{\text {Dr}} = H_{\text {MSDSE}} + \lambda _4 \text {ReLU} \big (D^{-\frac{1}{2}}A^{\text {Dr}}D^{-\frac{1}{2}}H_{\text {MSDSE}} \Theta ^g \big ), \end{aligned}$$

where $\lambda _4$ is a learnable parameter, *D* is the diagonal degree matrix of $A^{\text {Dr}}$, and $\Theta ^{g}$ is the learnable weight matrix for the graph convolutional layer.

For side effect features, we utilize the previously constructed $H^{\text {SE}}$ in Eq. (4) as our rudimentary representations. However, recognizing that drug and side effect features are constructed independently and may reside in different embedding domains, we employ a strategy to bridge this gap. To enhance side effect representations and align them with the richer drug embedding space, we introduce an adaptive residual strategy for side effects. Here, we generate learnable side effect embeddings $H_{\text {rand}}^{\text {SE}}$ as standardized random vectors (mean = 0, standard deviation = 0.1) and enhance it by incorporating HiGCN. We then define the composite side effect representation as:

7 $$\begin{aligned} \hat{H}^{\text {SE}} = \text {HiGCN} \big (A^{\text {SE}}, H_{\text {rand}}^{\text {SE}}, K^{\text {SE}}, \lambda _5 \big ) + \gamma \odot H^{\text {SE}} \end{aligned}$$

where $K^{\text {SE}} = 5$, *γ* is a 512-dimensional learnable vector, and the *⊙* symbol denotes element-wise multiplication. This operation scales each column of $H^{\text {SE}}$ by the corresponding element in *γ*, allowing us to adaptively re-weight and fine-tune the influence of the initial side effect features. $H_{\text {rand}}^{\text {SE}}$ is specifically trained to project $\hat{H}^{\text {SE}}$ into the same embedding domain as $\hat{H}^{\text {Dr}}$. This dual-path framework ensures cross-modal compatibility, allowing for more effective prediction models.

#### Drug-side effect prediction and optimization

This section outlines the prediction and optimization strategies for PromptSE and PromptSE+ models. For each drug-side effect pair $(d_i, s_j)$, previously defined embeddings are integrated to predict associations.

In PromptSE, drug $d_i$ is embedded as $h_i^{\text {Dr}}$, the *i*-th row of $H^{\text {Dr}}$ (Eq. 5), and side effect $s_j$ is embedded as $h_j^{\text {SE}}$, the *j*-th row of $H^{\text {SE}}$ (Eq. 4). In PromptSE+, these are $\hat{h}_i^{\text {Dr}}$, the *i*-th row of $\hat{H}^{\text {Dr}}$ (Eq. 6), and $\hat{h}_j^{\text {SE}}$, the *j*-th row of $\hat{H}^{\text {SE}}$ (Eq. 7). Predicted association likelihood are:

$$\begin{aligned} p_{ij} = h_i^{\text {Dr}} \cdot h_j^{\text {SE}}, \quad \hat{p}_{ij} = \hat{h}_i^{\text {Dr}} \cdot \hat{h}_j^{\text {SE}}. \end{aligned}$$

These likelihood $p_{ij}$ and $\hat{p}_{ij}$, with ground truth labels $y_{ij}$, are fed into a binary cross-entropy loss:

$$\begin{aligned} L = -\sum _{(d_i, s_j) \in S_{\text {train}}}\left[ y_{ij}\log (\sigma (p_{ij}))+(1-y_{ij})\log (1-\sigma (p_{ij}))\right] , \end{aligned}$$

where $\sigma (\cdot )$ is the sigmoid function. Parameters are optimized using Adam. Drug representation $H_{\text {MSDSE}}$ is initialized per Yu et al. ^10^, parameters $\lambda _1, \ldots , \lambda _5$ are initialized to 1, and *γ* and *Θ* (regardless of subscripts) are randomly initialized.

To prevent overfitting, early stopping is applied on the validation set, terminating training after 200 epochs without improvement. The Adam optimizer is used with a learning rate of $5\times 10^{-4}$ and a weight decay of $10^{-4}$, which are selected from $\{10^{-3},\,5\times 10^{-4},\,10^{-4},\,5\times 10^{-5},\,10^{-5}\}$ based on validation performance. The classification threshold is selected to be 0.5, determining positive or negative predictions. Model performance is evaluated on the test set using this threshold. Models are implemented in PyTorch and trained on an Nvidia GeForce RTX 4090 GPU with 16GB memory.

## Supplementary Information

Supplementary Information.

## Data Availability

To ensure reproducibility, data and code will be made publicly available at https://github.com/wanghao-1/PromptSE/tree/master.
