# Supplementary material for: PromptSE: drug side effect prediction with LLM-derived pharmacological representations
Source: Sci Rep. 2026 May 30;16:24812. doi: 10.1038/s41598-026-55667-7 (PMC13458511; doi:10.1038/s41598-026-55667-7)
Supplement: Supplementary file 1 [file 41598_2026_55667_MOESM1_ESM.pdf]

# Supplementary Document of "PromptSE: Drug Side Effect Prediction with LLM-Derived Pharmacological Representations"

## 1 Prompts for multi-stage LLM

This section presents the prompts used in the multi-stage LLM pipeline of our framework. For clarity, we report the actual prompt templates, placeholder variables, outputs with parsing procedures when applicable, and provide illustrative examples for Prompts C–E.

**Prompt A for Entity Characterization**

**Placeholder description.**
{side_effect}: the name of side effect $s_j$;
{tree_no}: the MeSH Tree Number of side effect $s_j$.

**Prompt template.**

> You will serve as an expert in pharmacology. Please help me provide definition of {side_effect} based on its MeSH Tree Number {tree_no}.
> Requirements:
> Please answer in English and avoid using abbreviations in the output.

**Output.**
The API returns a plain-text description of the queried side effect, denoted by $\alpha_j$.

**Prompt B for Entity Characterization**

**Placeholder description.**
{drug}: the name of drug $d_i$.

**Prompt template.**

> You will serve as an expert in pharmacology who helps me provide drug-target interactions of {drug}, so that I can infer what types of side effects would occur when {drug} is used, based on the knowledge you provide.
> Requirements:
> 1. Please answer in English and avoid using abbreviations in the output.
> 2. Do not specify the names of the targets; instead, mention the categories of the targets.

**Output.**
The API returns a plain-text summary of the drug-target interactions, denoted by $t_i$.

Prompt C for Entity Characterization

**Placeholder description.**
{drug}: the name of drug $d_i$;
{interactions}: the drug-target interactions $t_i$ obtained from Prompt B.

**Prompt template.**

You will serve as an expert in pharmacology to provide information about {drug}, covering:
1. Indicate the administration route of {drug}: oral, intravenous, intramuscular, subcutaneous, buccal, rectal, ophthalmic, nasal, topical or a combination of these.
2. Analyze the anatomical location where {drug} exerts its action, including the involved systems and organs.
3. Identify the distribution of {drug}'s targets (including the involved systems and organs), based on the provided drug-target interactions: {interactions}.
4. Determine which structural or toxicological features of {drug} could lead to side effects, including functional groups, stereochemistry, lipophilicity/hydrophilicity, and metabolites.
5. Explain the metabolic pathways of {drug}, including the involved systems and organs, enzymes, and metabolites formed.
Output the results in the following JSON format:
{
"routes of administration": "Administration routes of {drug}",
"sites of action": "Anatomical location of action for {drug}",
"distribution of targets": "Distribution of {drug}'s targets",
"structural features": "Structural or toxicological features of {drug} that may cause side effects",
"metabolic pathways": "Metabolic pathways of {drug}"
}.
Requirements:
1. Respond in English.
2. If any information does not exist, use None to indicate it.
3. Do not return any information outside of the JSON.
4. When answering regarding systems, use one of the following: "musculoskeletal system, circulatory system, respiratory system, digestive system, urinary system, nervous system, endocrine system, reproductive system".

**Output and parsing.**
The API returns a raw string whose main content is a JSON-formatted payload. In our pipeline, we remove surrounding quotation marks, Markdown formatting markers, and extra line breaks before extracting the inner JSON content for downstream use. The resulting drug property profile is denoted by $\beta_i$.

**Example.**

For example, when {drug} = Fentanyl and {interactions} = ["nervous system", "immune system"], the parsed output is of the following form:
{
"routes of administration": ["intravenous", "intramuscular" . . . ],
"sites of action": "central nervous system, peripheral nervous system",
"distribution of targets": ["nervous system", "immune system"],
"structural features": "high lipophilicity, phenylpiperidine scaffold, . . . ",
"metabolic pathways": "primarily hepatic metabolism via CYP3A4/3A5, . . . "
}.

## Prompt D for Mechanism-oriented Inference

**Placeholder description.**
{drug}: the name of drug $d_i$;
{description}: the drug description $\beta_i$ obtained from Prompt C;
{side_effect}: the name of side effect $s_j$;
{definition}: the side effect description $\alpha_j$ obtained from Prompt A.

**Prompt template.**

Please analyze how the drug {drug} with the description "{description}" causes the side effect {side_effect} with the definition "{definition}", from the following four categories: administration route, metabolism pathway, target selectivity, and structural properties.
Your answer should include the following information:
1. The **category** that is the cause (one of the four listed above).
2. A concise **explanation** of how this category leads to the side effect, less than 30 words:
   - If the cause is the administration route, specify the exact route of administration and the involved organs or systems.
   - If the cause is the metabolism pathway, specify which organ is responsible for metabolism and what substances are being metabolized.
   - If the cause is target selectivity, specify the location of the target distribution.
   - If the cause is structural properties, specify the exact structure and characteristics.
   - Choose the one reason that you think is the most likely.
3. The **summary** of the reasoning behind the above answer.

Requirements:
1. Your response should be in JSON format, with the following keys: category, explanation and summary.
2. Do not return any information outside of the JSON.
3. The answer should avoid redundant information, ensuring high information density.
4. Answer in English.

**Output and parsing.**
As in Prompt C, the API returns a raw string whose main content is is in JSON format. We apply the same post-processing procedure to extract the inner JSON content for downstream use, denoted by $c_{ij} = f(d_i, \beta_i, s_j, \alpha_j)$.

**Example.**

For example, when {drug} = Gatifloxacin, {side_effect} = Conjunctival cyst,
{description} = {\n "routes of administration": [ophthalmic, ... \n} and
{definition} = "Conjunctival cyst refers to ... ",
the parsed output is of the following form:
{
"category": "administration route",
"explanation": "Ophthalmic administration delivers gatifloxacin directly to the conjunctiva, causing local epithelial irritation or cystic metaplasia in susceptible individuals.",
"summary": "Conjunctival cyst is a localized ocular adverse effect most plausibly triggered by direct exposure from ophthalmic formulation, not systemic exposure, metabolism, off-target binding, or structural features alone."
}.

## Prompt E for Profile Summarization

**Placeholder description.**
{side_effect}: the name of side effect $s_j$;
{definition}: the side effect description $\alpha_j$ obtained from Prompt A;
{listed_exps}: the list of mechanism-oriented inferences $\{(d_i, c_{ij}) : i \in \tilde{I}_j\}$ derived from Prompt D.

**Prompt template.**

Please summarize the causes of the side effect "{side_effect}" (defined as: "{definition}") based on the provided explanations of how specific drugs cause "{side_effect}": "{listed_exps}".
The causes fall into four categories: administration route, metabolism pathway, target selectivity, and structural properties. Categorize each cause and provide a concise summary per category:
- If the cause is **administration route**, list only the specific route (e.g., Ophthalmic) causing "{side_effect}" (max 5 words). Do not include additional explanations or effects.
- If the cause is **metabolism pathway**, summarize the metabolic organs/systems and the types of metabolites (e.g., Liver, Oxidative Products) (max 15 words). Use specific metabolite categories rather than specific metabolite names.
- If the cause is **target selectivity**, summarize the receptor types and their locations (organ/system) (e.g., Adrenergic Receptor, digestive system) (max 15 words). Do not include specific receptor names.
- If the cause is **structural properties**, summarize the drug's structural features (e.g., Lipophilicity) (max 10 words).
- If any category is absent, respond with "None".
- If how the drug causes "{side_effect}" is unclear, guess the most likely cause based on the "{definition}".
Requirements:
1. Only include factors contributing to "{side_effect}".
2. Your response must be in JSON format with the keys: "administration route", "metabolism pathway", "target selectivity", and "structural properties".
3. Do not include any information outside of the JSON.
4. Identify at least one cause for "{side_effect}".
5. Avoid redundancy and ensure high information density.
6. Respond in English and avoid abbreviations.
7. When answering regarding systems, use one of the following: "musculoskeletal system, circulatory system, respiratory system, digestive system, urinary system, nervous system, endocrine system, reproductive system".

**Output and parsing.**
As in Prompt C, the API returns a raw string whose main content is is in JSON format. We apply the same post-processing procedure to extract the inner JSON content for downstream use, denoted by $p_j = \left(p_j^{(1)}, p_j^{(2)}, p_j^{(3)}, p_j^{(4)}\right)$.

**Example.**

For example, when {side_effect} = Conjunctival cyst, {definition} = "Conjunctival cyst refers to . . . ", and {listed_exps} = [["Gatifloxacin", "administration route: Ophthalmic administration delivers . . . "], . . . ], the parsed output is of the following form:

{
"administration route": "Ophthalmic",
"metabolism pathway": "None",
"target selectivity": "None",
"structural properties": "None"
}.

## 2 Formulas for the evalution metrics

This section provides the specific formulas for the evaluation metrics used in the experiments: Kolmogorov-Smirnov (KS) test, Area Under the Precision-Recall Curve (AUPR), Area Under the Receiver Operating Characteristic Curve (AUC), Macro-averaged F1 score (Macro-F1) and Matthews Correlation Coefficient (MCC).

The KS test is used to determine whether the distributions of two sample sets are identical. Let the two sample sets be $\{X_1, X_2, \ldots, X_n\}$ and $\{Y_1, Y_2, \ldots, Y_m\}$, with sample sizes $n$ and $m$, respectively. Their empirical distribution functions are defined as:

$$F_n(x) = \frac{1}{n}\sum_{i=1}^{n} I(X_i \leq x), \quad G_m(x) = \frac{1}{m}\sum_{j=1}^{m} I(Y_j \leq x).$$

The KS statistic $D_{n,m}$ is defined as:

$$D_{n,m} = \sup_{x\in\mathbb{R}} |F_n(x) - G_m(x)|.$$

The KS statistic ranges from 0 to 1, with larger values indicating greater differences between the two distributions.

AUPR quantifies the trade-off between precision and recall across different classification thresholds, which is calculated as the area under the curve of precision versus recall:

$$\text{AUPR} = \int_0^1 \text{Precision}(t)\, d\text{Recall}(t),$$

where $\text{Precision}(t) = \frac{\text{TP}(t)}{\text{TP}(t)+\text{FP}(t)}$ and $\text{Recall}(t) = \frac{\text{TP}(t)}{\text{TP}(t)+\text{FN}(t)}$ at threshold $t$. The Area Under the Precision-Recall Curve (AUPR) ranges from 0 to 1, with larger values indicating better model performance in balancing precision and recall, especially for imbalanced datasets.

AUC measures the model's ability to distinguish between positive and negative classes, which is computed as:

$$\text{AUC} = \int_0^1 \text{TPR}(t)\, d\text{FPR}(t),$$

where $\text{TPR}(t) = \frac{\text{TP}(t)}{\text{TP}(t)+\text{FN}(t)}$ and $\text{FPR}(t) = \frac{\text{FP}(t)}{\text{FP}(t)+\text{TN}(t)}$. AUC ranges from 0 to 1, with larger values indicating better model performance in distinguishing between positive and negative classes.

The Macro-F1, the unweighted mean of the F1-scores of different classes, is given by:

$$\text{Macro-F1} = \frac{1}{2}\left(\text{F1}_+ + \text{F1}_-\right),$$

where $\text{F1}_+$ and $\text{F1}_-$ denote the F1-scores of the positive and negative classes, respectively. Macro-F1 ranges from 0 to 1, with larger values indicating better balanced performance across classes.

MCC balances all confusion matrix elements defined as:

$$\text{MCC} = \frac{\text{TP}\cdot\text{TN} - \text{FP}\cdot\text{FN}}{\sqrt{(\text{TP}+\text{FP})(\text{TP}+\text{FN})(\text{TN}+\text{FP})(\text{TN}+\text{FN})}}.$$

For all evaluated metrics, a value closer to 1 indicates more accurate predictions. The calculation of F1-score and MCC requires a specific threshold $t$. MCC ranges from -1 to 1, with larger values indicating better classification performance, balancing true positives, true negatives, false positives, and false negatives.
